# Web-Based Application for Cognitive and Functional Assessments in Dementia Screening: Mixed Methods, User-Centered Development Approach

**DOI:** 10.2196/85454

**Published:** 2026-02-19

**Authors:** Nattaporn Piyaamornpan, Suwat Srisuwannanukorn, Kosit Tangthamrongthanawat, Pornsawan Mekhasingharak, Chatchawan Rattanabannakit, Saowalak Hunnangkul, Natthamon Wongkom, Vorapun Senanarong

**Affiliations:** 1 Division of Neurology, Department of Medicine, Faculty of Medicine Siriraj Hospital, Mahidol University, 2 Wang Lang Rd, Siriraj, Bangkok Noi, Bangkok, 10700, Thailand, 66 24197665, 66 24123009; 2 Department of Medicine, Phatthalung Hospital, Phatthalung, Thailand; 3 Division of Neurology, Department of Internal Medicine, Tha Sala Hospital, Nakhon Si Thammarat, Thailand; 4 Division of Neurology, Department of Medicine, Faculty of Medicine Vajira Hospital, Navamindradhiraj University, Bangkok, Thailand; 5 Department of Internal Medicine, Nakhon Phanom Hospital, Nakhon Phanom, Thailand; 6 Regional Health Promotion Center 2 Phitsanulok, Phitsanulok, Thailand; 7 Department of Research and Development, Faculty of Medicine Siriraj Hospital, Mahidol University, Bangkok, Thailand

**Keywords:** Alzheimer disease, cognitive screening, dementia, functional assessment, neurocognitive disorders, neuropsychology, web application

## Abstract

**Background:**

Digital health technologies offer new opportunities for cognitive screening and monitoring among older adults. In Thailand, where dementia prevalence is rising, accessible web-based cognitive tools remain limited despite their potential to facilitate early detection and community-based assessment. Understanding usability and validity is critical to ensure successful implementation in real-world contexts.

**Objective:**

This study aimed to develop and validate a web-based application, *Healthy Brain Test*, for cognitive and functional assessments in dementia screening among older Thai adults. Specific objectives were to (1) design user-centered cognitive modules covering key cognitive domains and (2) evaluate correlations between the web-based assessments and conventional clinical tools to determine diagnostic cutoffs for cognitive impairment.

**Methods:**

We designed *Healthy Brain Test* as a self-administered web application suitable for older users and their caregivers. The platform includes digital versions of the Thai Mental State Examination (e-TMSE), a clock drawing test, and a category verbal fluency test, along with electronic versions of the short form of the Informant Questionnaire on Cognitive Decline in the Elderly (IQCODE-16) and cognitive instrumental activities of daily living (IADLs). Participants completed both web-based and paper-based assessments. Correlations between modalities were analyzed, and receiver operating characteristic (ROC) curves were generated to determine sensitivity and specificity. Data were analyzed using SPSS for Windows, version 30.0 (IBM Corp) and MedCalc Statistical Software (MedCalc Software Ltd).

**Results:**

A total of 198 older adults participated (women: 137/198, 69.2%; median age 69.4 years), with 57.1% (113/198) having more than 6 years of education. Of the 198 participants, 44 were diagnosed with major neurocognitive disorder, 58 were diagnosed with mild neurocognitive disorder, and 96 were cognitively normal. The e-TMSE showed strong agreement with the traditional TMSE (*r*=0.837; *P*<.001). Category verbal fluency, IQCODE-16, and IADL modules also demonstrated significant correlations (*P*<.001). The e-TMSE achieved an area under the ROC curve of 0.84 (bootstrapped 95% CI 0.78‐0.89); a cutoff ≤23 provided 88.6% sensitivity and 70.1% specificity for identifying major neurocognitive disorder. Participants reported high ease of use and engagement during pilot testing.

**Conclusions:**

*Healthy Brain Test* demonstrated strong validity and usability as a web-based cognitive and functional assessment platform for dementia screening. Its integration of established cognitive measures into a digital interface enables remote, accessible, and user-friendly evaluation for older adults and caregivers. Future research should assess long-term feasibility, user adherence, and integration with clinical workflows to support large-scale screening initiatives.

## Introduction

Cognitive performance often declines with normal healthy aging [1], with measurable deficits in episodic memory, working memory, verbal fluency, and executive control processes such as visual attention and set-shifting [2]. These age-related changes do not inevitably progress to dementia. However, dementia remains a significant public health challenge in Thailand, and prevalence rises sharply with age. A national survey reported that 3.3% of Thai adults aged 60 years and older have some form of dementia, increasing from 1% at ages 60 years to 64 years to 31.3% among those aged 90 years and older [3].

Digital technologies have advanced cognitive screening, offering potential benefits for clinical treatment, therapeutic interventions, and behavioral research. Web-based applications now extend beyond screening to support self-management, caregiver education, cognitive rehabilitation, and remote clinical monitoring. Recent studies indicate that such applications can contribute across the dementia care continuum [4-8].

In Thailand, web-based dementia care applications are still early in development. *Dementia U-Care*, designed to help community health workers screen and monitor cognitive data, has demonstrated feasibility and positive user experiences [9]. Vorasut and Tantatsanawong [10] introduced smartphone-based screening tools using the Thai version of the Mini-Mental State Examination (MMSE; *MMSE-Thai 2002*) with text-to-speech and audio recording. More recently, Yangyuensathaporn et al [11] validated the Application-Based Cognitive Screening Test for older Thai adults, reporting 92.9% sensitivity and 70% specificity.

Standard neuropsychological instruments, including the MMSE and Montreal Cognitive Assessment (MoCA), have long served as benchmarks for detecting early cognitive impairment. The MMSE has a sensitivity of 87% to 97% and specificity of 70% to 82% depending on the population and selected cutoff points [12]. The MoCA, designed to be more sensitive to mild cognitive impairment, typically achieves about 90% sensitivity and approximately 87% specificity [13].

Given population aging and the demand for accessible screening, there is a need to develop localized, culturally appropriate digital tools. Therefore, we designed *Healthy Brain Test*, a test battery usable via a web-based application on tablet computers and mobile phones. Our objectives were to develop a web-based cognitive and functional assessment platform aimed at dementia screening in older Thai adults and to design diverse cognitive modules targeting different cognitive domains. We also sought to evaluate correlations between the web-based assessments and traditional tools.

## Methods

### Study Population

Participants older than 40 years were prospectively and purposefully recruited from the Faculty of Medicine Siriraj Hospital, Mahidol University, Bangkok, Thailand. Recruitment aimed to evaluate cognitive and functional performance for dementia screening using a dedicated web-based application. All participants had either relatives or long-term caregivers who could provide relevant information on cognitive status and daily functioning at present and over the past 10 years.

### Inclusion and Exclusion Criteria

The inclusion criteria were provision of informed consent after full explanation of study objectives and procedures; adequate literacy, vision, and hearing; and ability to read and comprehend Thai. The exclusion criteria were acute confusion, impaired decision-making capacity due to cognitive or medical conditions, and severe physical or psychiatric illness.

### Diagnostic Classification

The diagnoses of major and mild neurocognitive disorders were established according to the *Diagnostic and Statistical Manual of Mental Disorders, Fifth Edition, Text Revision* (*DSM-V-TR*) criteria [14]. Cognitively healthy control participants were recruited from relatives or spouses of patients and community volunteers. These individuals had no reported cognitive decline, demonstrated normal performance on cognitive assessments, and maintained independence in daily functioning.

### Ethical Considerations

All participants provided written informed consent before enrollment. The study protocol was approved by the Human Research Protection Unit, Faculty of Medicine Siriraj Hospital, Mahidol University (COA No. Si 982/2021), and conducted in accordance with the Declaration of Helsinki. All participant data were kept confidential and anonymized in accordance with the approved ethical protocol. Participants in Phase I received ฿100 (US $3.20) for their participation, while participants in Phase II did not receive any financial compensation.

### Study Design

This study used a user-centered design approach to develop and validate a web-based cognitive and functional screening application for the early detection of dementia. Development proceeded through 3 consecutive phases involving collaboration among computer scientists, health informaticians, psychologists, and neurologists. All phases were guided by clinical relevance, usability, and data security considerations.

### Phase I: Prototype Development and Usability Testing

In Phase I, we developed a prototype of the *Healthy Brain Test* web-based application and iteratively refined it to optimize usability. The application was designed for Android-based systems to ensure widespread accessibility and compatibility with mobile devices commonly used in community and clinical settings. Usability testing sessions with volunteer participants generated feedback that informed successive iterations of the user interface. The interface comprised 2 sections. The home page introduced the application, provided general usage instructions, and presented an electronic informed consent form. Users were required to review and agree to the consent before accessing the assessment modules. The assessment section included 5 digital cognitive and functional tasks adapted from established neuropsychological assessments. Each task was preceded by a brief, standardized instructional message, and the design enhanced interactivity while reducing reliance on motor skills. Initial trials were conducted with volunteer participants to ensure content comprehension, ease of use, and engagement.

### Phase II: The Pilot Study

A pilot study at Siriraj Hospital, Thailand, evaluated the validity of the *Healthy Brain Test* web-based cognitive screening application against traditional paper-and-pencil assessments. A total of 77 participants were enrolled (50 women and 27 men). Participants were categorized into 3 groups: 21 with major neurocognitive disorder (mild to moderate dementia), 23 with mild cognitive impairment, and 33 cognitively normal control participants. Each participant completed both conventional and digital cognitive assessments. The traditional paper-and-pencil assessments included the standard Thai Mental State Examination (TMSE) and the clock drawing test (CDT), administered under the supervision of trained clinicians. After the paper-and-pencil tests, participants were guided through the web-based *Healthy Brain Test* application. The full assessment session lasted approximately 1 hour to 2 hours depending on participant performance.

### Phase III: Practical Use and Data Collection

In the final phase, we implemented the *Healthy Brain Test* web-based application in real-world settings across 5 health centers in Thailand. The sites were Siriraj Hospital, Vajira Hospital, Phatthalung Hospital, Phitsanulok Health Center 2, and Nakhon Phanom Hospital. This phase aimed to assess practical use in community-based and clinical environments and to facilitate the early detection of cognitive impairment and dementia. The application was accessible via health care professionals or self-assessment under observation, and participants could complete the digital screening autonomously or with minimal assistance. This approach supported broader reach and feasibility across diverse settings, including rural areas. We enrolled 198 participants (137 women and 61 men). Based on clinical evaluation, participants were categorized as follows: 44 with major neurocognitive disorder, 58 with mild cognitive impairment, and 96 with normal cognition. Digital screening assessed cognitive and functional performance using 5 standardized tasks embedded in the application. We also collected demographic and educational data and stored them on secure cloud servers, ensuring confidentiality and compliance with data protection standards.

### The Healthy Brain Test Web-Based Application

**Figure 1.**
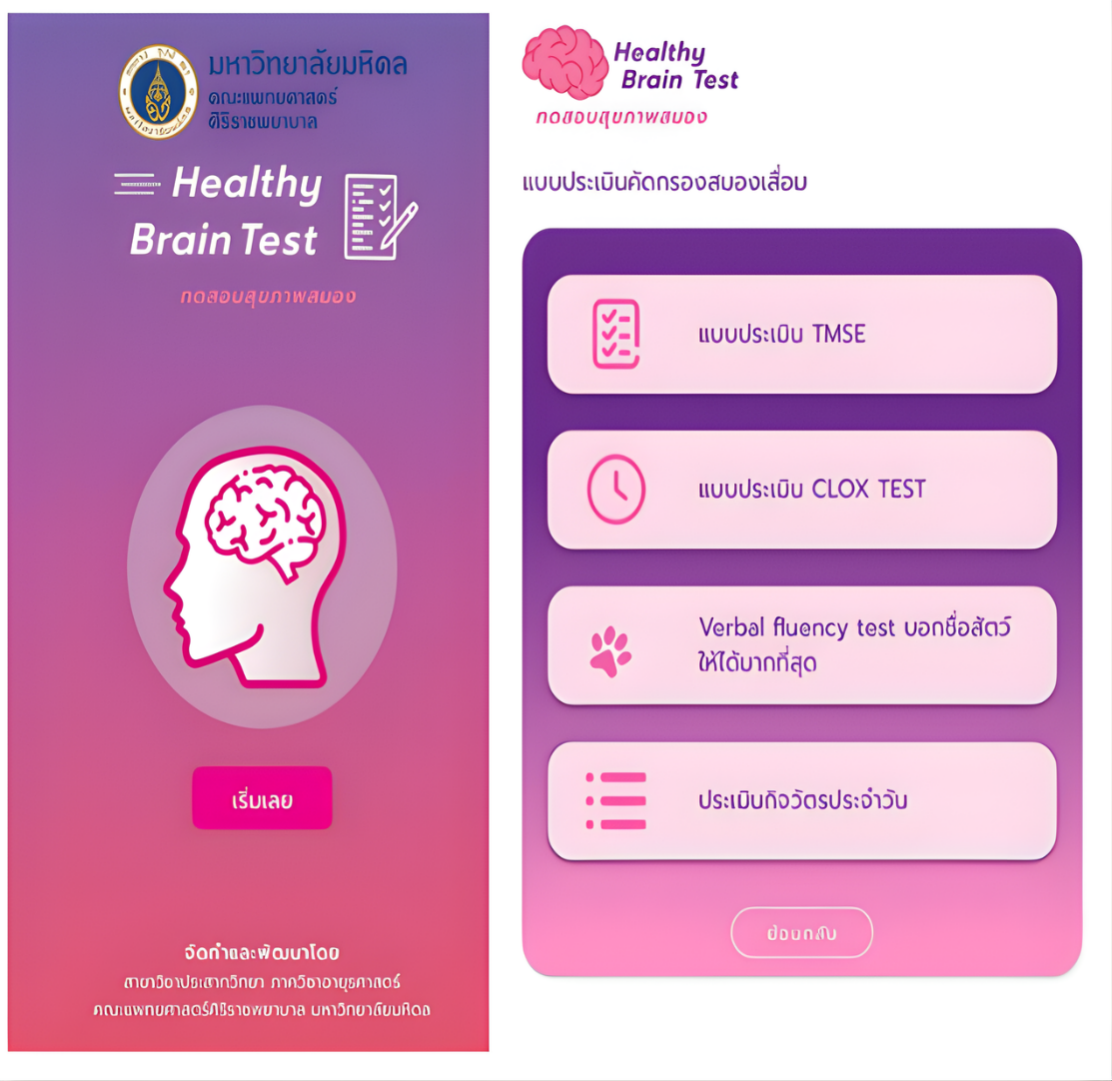
Healthy brain test module overview illustrating the list of 5 modules: electronic Thai Mental State Examination (e-TMSE), the Clox test (digitized clock drawing), category verbal fluency test (animal naming), short form of the Informant Questionnaire on Cognitive Decline in the Elderly (IQCODE-16), and cognitive instrumental activities of daily living (cognitive IADLs).

*Healthy Brain Test* is a web-based digital cognitive and functional screening tool that integrates 5 core neuropsychological tasks digitally adapted from validated conventional tests widely used to assess cognitive function. The battery included the following 5 tasks: a slightly modified electronic TMSE (e-TMSE); the CDT implemented as the Clox test; and a category verbal fluency test (VFT). The remaining tasks were the short form of the Informant Questionnaire on Cognitive Decline in the Elderly (IQCODE-16) and cognitive instrumental activities of daily living (cognitive IADLs). Illustrative screenshots of the assessment modules are shown in Figure 1.

### Electronic Thai Mental State Examination

**Figure 2.**
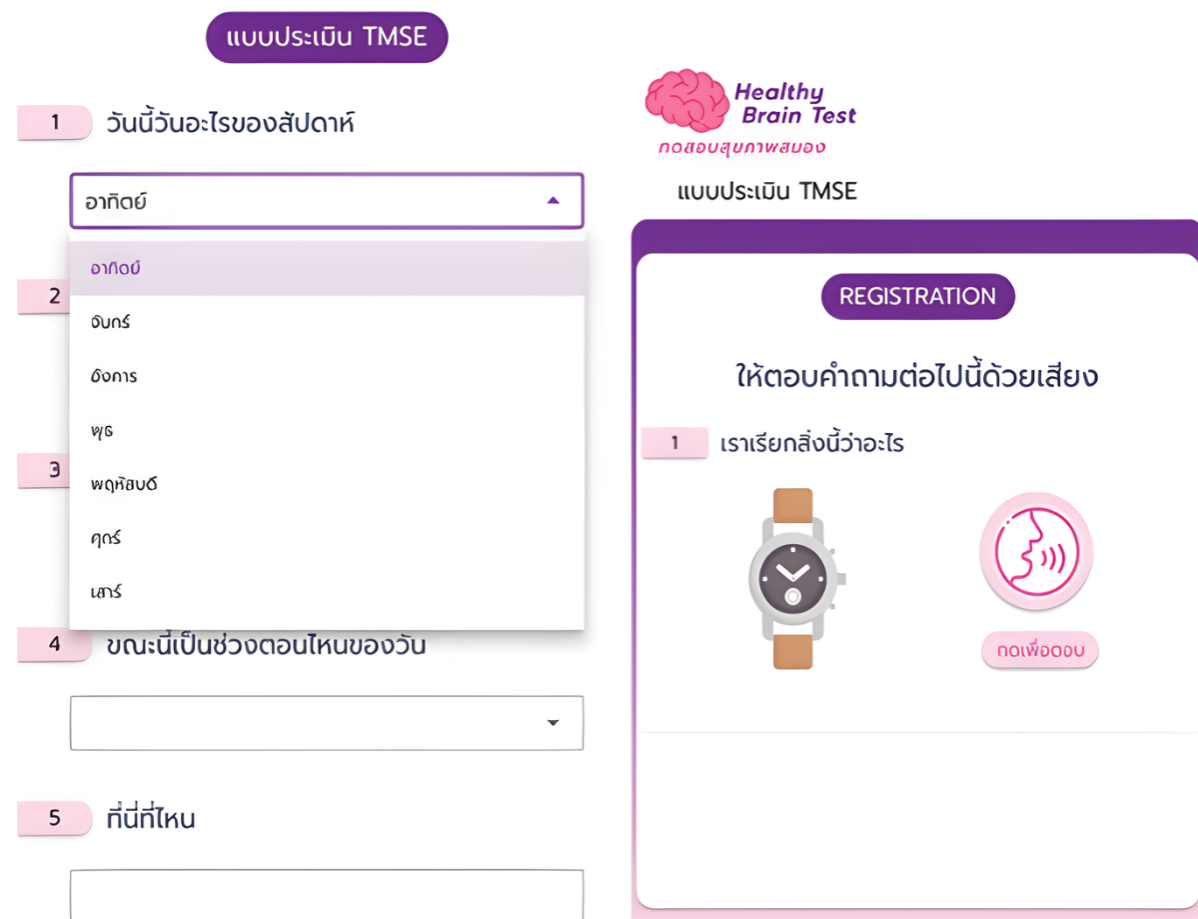
Electronic Thai Mental State Examination (e-TMSE) sample screens showing orientation and registration, which use touch-based entry.

The e-TMSE, a digital adaptation of the standard paper-and-pencil TMSE [15], was designed to assess cognitive function through an interactive web-based interface. The traditional TMSE consists of 11 items covering 6 cognitive domains: orientation, registration, attention and calculation, recall, language, and visuospatial abilities. Each component was implemented in the digital platform, and the original maximum total score of 30 points was preserved. Participants used touch to choose from on-screen options or to handwrite responses for language items. Samples of the e-TMSE test screens are shown in Figures2  3.

**Figure 3.**
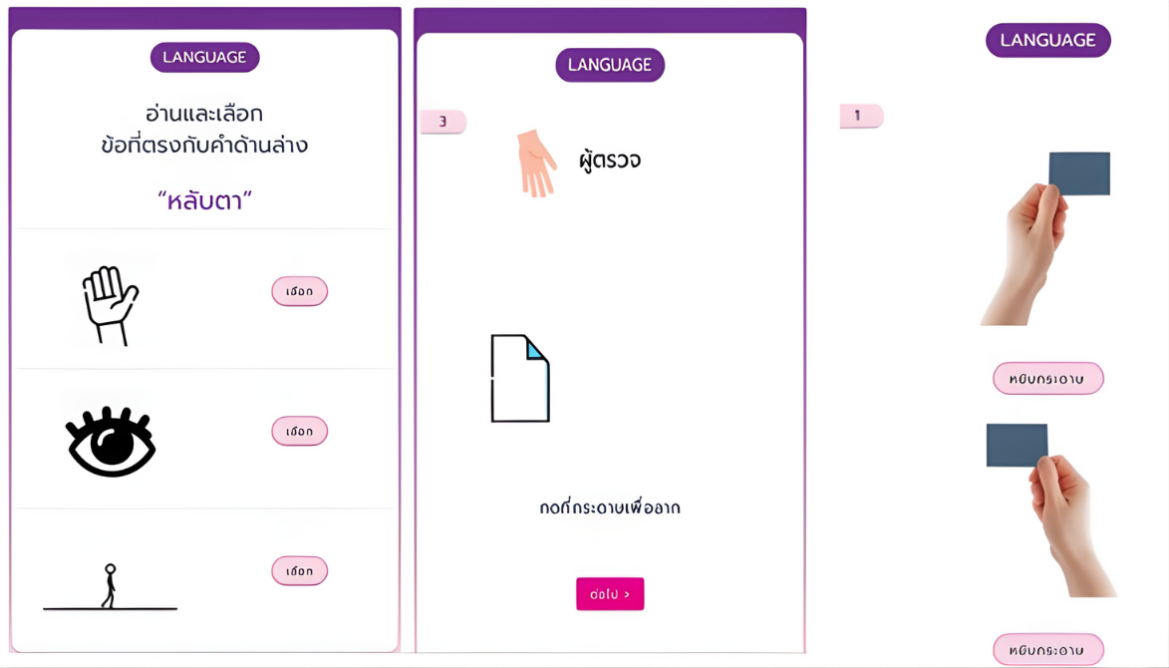
Electronic Thai Mental State Examination (e-TMSE) sample screens showing the language items, which allow on-screen handwriting and choice selection.

### Clock Drawing Test

**Figure 4.**
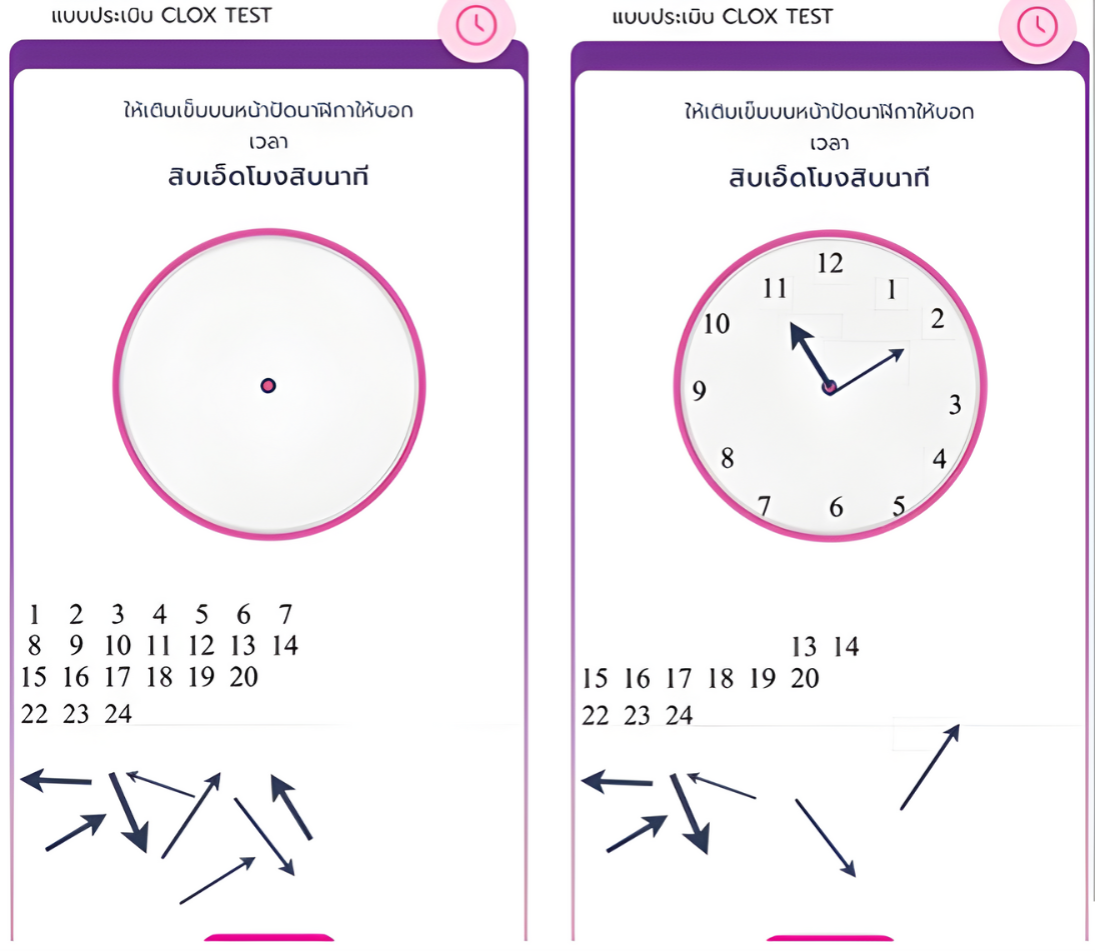
Digitized clock drawing (the clox test) sample screens showing how participants draw a clock showing a specified time on a touch-based canvas.

The CDT is widely used to assess visuospatial ability and executive function and has attracted interest for early cognitive impairment screening [16]. In this study, a digitized CDT variant, the Clox test, was implemented within the *Healthy Brain Test* application. Participants drew a clock showing a specified time on a touch-based interface. The test used a 10-point scale, where 0 indicated severe impairment and 10 represented normal performance. Illustrative screenshots are shown in Figure 4.

### Category VFT

**Figure 5.**
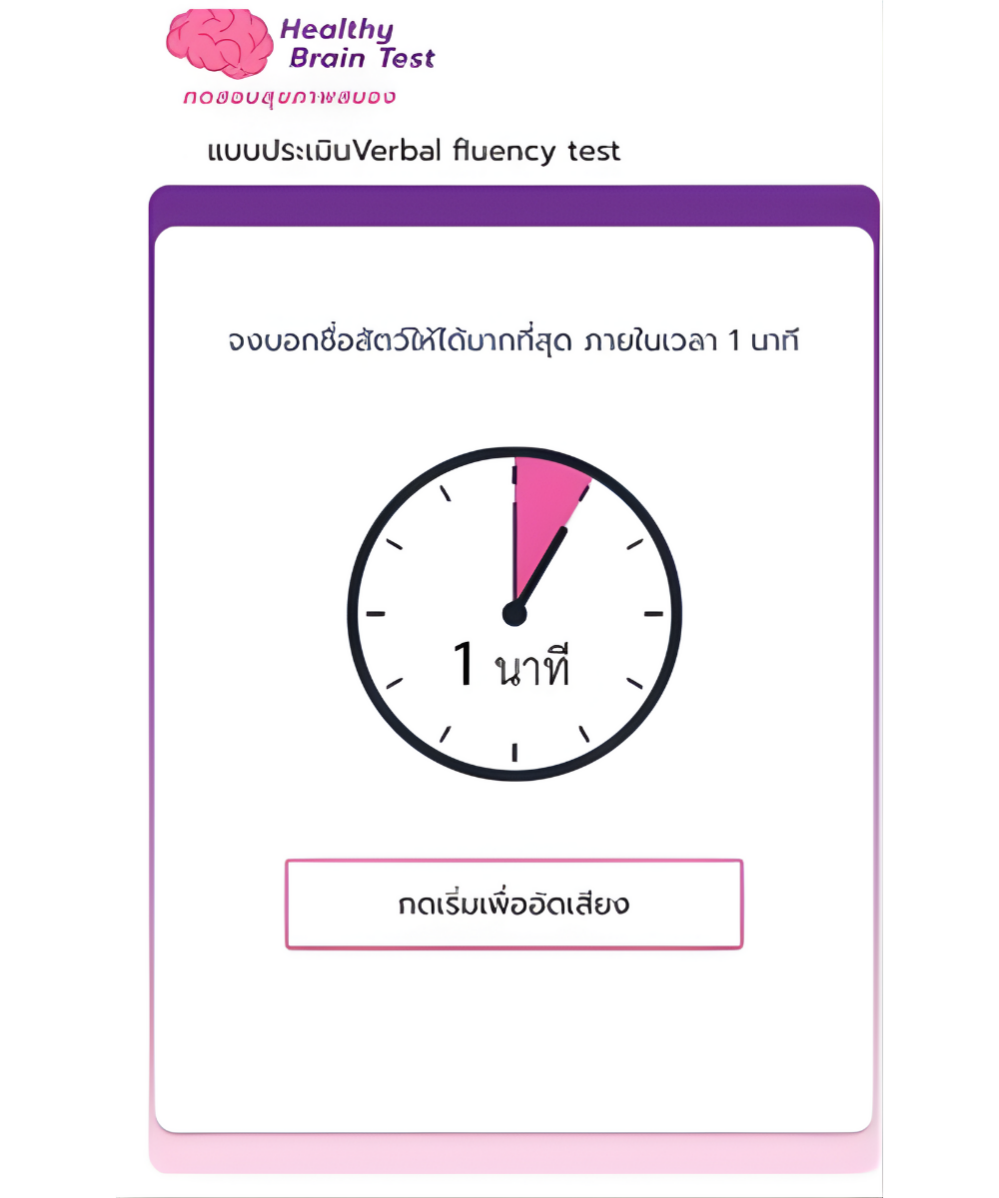
Category verbal fluency test (animal naming) sample screens, in which participants name as many animals as possible in 1 minute. The interface provides standardized instructions and timed entry.

The category (semantic) VFT is a well-established neuropsychological measure that assesses executive function and semantic memory and shows high sensitivity for detecting cognitive impairment, including mild cognitive impairment and dementia [17,18]. In this task, participants generate as many words as possible from a specified category within a limited time, typically 1 minute. In this study, we used the animal naming category and instructed participants to name as many animals as possible within 1 minute. Illustrative screenshots are shown in Figure 5.

### Short Form of the Informant Questionnaire on Cognitive Decline in the Elderly

The short form of the IQCODE consists of 16 items that assess informants’ perceptions of a participant’s cognitive decline by comparing current performance with performance 10 years earlier. In this study, informants were relatives, long-term paid caregivers, or neighbors who had been providing some care. They rated change on a 5-point scale from 1 (much improved) to 5 (much worse), and the score was the mean of the 16 items [19].

### Cognitive IADLs

The cognitive IADL module in *Healthy Brain Test* is derived from the validated Thai Activities of Daily Living (ADL) scale [20]. The instrument includes 6 basic ADL items and 7 cognitive IADL items, and it evaluates the capacity to perform essential daily tasks that require physical function and cognitive integration. The Thai ADL measure has shown strong psychometric properties, including high test-retest reliability and robust construct validity. It also shows strong correlations with the TMSE, Clinical Dementia Rating, Barthel Index, and Functional Activities Questionnaire.

### Measurements

#### Baseline Assessment

Trained physicians and neurologists conducted structured interviews to obtain baseline clinical and demographic data, including age, sex, educational attainment, and cognitive status.

#### Statistical Analysis

We calculated the required sample size using an estimated dementia prevalence of 20% in older Thai adults based on national guidelines [21]. With 95% confidence and a 5% margin of error, we applied the standard prevalence formula:


$$n=\frac{Z^{2}\times P\times (1-P)}{d^{2}}$$

where *n* is the required sample size, *Z* is the 95% confidence *Z*-score (1.96), *P* is the expected prevalence (0.20), and *d* is the margin of error (0.05). This yielded a minimum sample size of 246 (5% margin of error) participants.

Although our planned sample size was 246 participants, the final sample comprised 198 participants. Precision is therefore slightly lower than planned; 95% CIs are reported for key estimates. For example, a 20% prevalence has a 95% margin of error of about 5.6% at n=198 versus 5.0% at n=246. However, if the web application for cognitive screening is good enough, the results should still be satisfactory. Moreover, in Thailand, medical services at general hospitals in the peripheral areas of the country are overloaded, causing investigators to be unable to complete sufficient case collection for this study. Data analysis was performed using SPSS version 30 (IBM Corp) and MedCalc Statistical Software (MedCalc Software Ltd). Descriptive statistics summarized baseline demographic and clinical characteristics. To assess validity, we computed Spearman correlation coefficients between digital test scores and conventional paper-and-pencil scores (TMSE and the CDT) because the data were not normally distributed. We considered *P*<.05 statistically significant. We also conducted receiver operating characteristic (ROC) curve analyses to evaluate the application’s ability to distinguish cognitively impaired individuals from cognitively normal control participants. We calculated the area under the curve (AUC) for each digital test domain to quantify overall diagnostic performance.

## Results

### Phase II: Participant Demographics and Initial Validation

This phase evaluated concordance and diagnostic accuracy between traditional paper-and-pencil assessments and web-based application tests. A total of 77 participants were categorized into 3 groups: those with dementia (n=21), those with mild cognitive impairment (n=23), and cognitively normal control participants (n=33). The dementia group had a significantly higher mean age than the other groups (*P*=.003). All cognitive and functional measures differed significantly between groups (all *P*<.001). The dementia group had the lowest performance on paper-and-pencil TMSE, e-TMSE, clock drawing, and verbal fluency, and they had the highest ADL, cognitive IADL, and IQCODE-16 scores, indicating greater functional impairment and cognitive decline. Demographic characteristics for all 77 participants are shown in Table 1.

**Table 1.** Participant demographics and clinical characteristics, initial phase (n=77).

Participant characteristics	Dementia (n=21)	MCI^a^ (n=23)	Normal cognition (n=33)	Total (n=77)	*P* value^b^
Age (years), median (IQR)	73.0 (69.5-79.0)	69.0 (63.0-75.0)	66.0 (56.5-72.0)	69.0 (63.0-75.0)	.003
Female, n (%)	14 (66.7)	15 (65.2)	22 (66.7)	51 (66.2)	.99
Formal education, n (%)					
Primary	13 (61.9)	6 (26.1)	5 (15.2)	24 (31.2)	
Secondary	1 (4.8)	4 (17.4)	4 (12.1)	9 (11.7)	
Vocational	3 (14.3)	3 (13)	5 (15.2)	11 (14.3)	
Bachelor’s degree	3 (14.3)	8 (34.8)	15 (45.5)	26 (33.8)	
Postgraduate	1 (4.8)	2 (8.7)	4 (12.1)	7 (9.1)	
e-TMSE^c^, median (IQR)	21.0 (16.5-23.0)	25.0 (24.0-28.0)	28.0 (25.5-29.0)	25.0 (23.0-28.0)	<.001
Clox^d^ test, median (IQR)	7.0 (1.5-8.0)	10.0 (9.0-10.0)	10.0 (9.5-10.0)	10.0 (7.0-10.0)	<.001
Verbal fluency test, mean (SD)	11.11 (4.99)	17.61 (6.80)	19.12 (4.61)	16.48 (6.35)	<.001
Basic ADLs^e^, median (IQR)	7.0 (3.5-9.0)	0.0 (0.0-1.0)	0.0 (0.0-0.0)	0.0 (0.0-2.5)	<.001
Cognitive IADLs^f^, median (IQR)	6.0 (3.5-8.5)	0.0 (0.0-1.0)	0.0 (0.0-0.0)	0.0 (0.0-2.0)	<.001
IQCODE-16^g^, median (IQR)	4.0 (3.8-4.6)	3.4 (3.2-3.7)	3.1 (3.0-3.3)	3.3 (3.1-3.8)	<.001
TMSE^h^, median (IQR)	22.0 (21.0-25.0)	28.0 (26.0-29.0)	28.0 (27.0-29.5)	27.0 (24.0-29.0)	<.001
Clock drawing test (paper), median (IQR)	6.0 (5.0-10.0)	10.0 (10.0-10.0)	10.0 (9.3-10.0)	10.0 (7.0-10.0)	<.001

a MCI: mild cognitive impairment.

b Between-group comparisons.

c e-TMSE: electronic Thai Mental State Examination.

d Clox: digitized clock drawing.

e ADLs: activities of daily living.

f IADLs: instrumental activities of daily living.

g IQCODE-16: 16-item Informant Questionnaire on Cognitive Decline in the Elderly.

h TMSE: Thai Mental State Examination.

### Digital and Standard Cognitive Screening Tests

The paper-and-pencil TMSE and e-TMSE had a strong, statistically significant correlation (Spearman *ρ*=0.837, *P*<.001), indicating that the digital version reflects the traditional assessment. The mean paper-and-pencil TMSE score was 26.04 (SD 3.87; median 27), while the mean e-TMSE score was 24.91 (SD 4.18; median 25). Subdomain analysis showed comparable performance across most cognitive components. The score distribution indicated a population predominantly comprising cognitively intact individuals and those with mild cognitive impairment. Details are provided in Table 2.

**Table 2.** Total and domain scores on the paper-and-pencil Thai Mental State Examination (TMSE) versus those of the electronic TMSE (e-TMSE).

TMSE components	Paper-and-pencil TMSE score, mean (SD; median)	e-TMSE score, mean (SD; median)	Correlation, Spearman ρ^a^	*P* value
Total TMSE score	26.04 (3.87; 27)	24.91 (4.18; 25)	0.837	.001
Orientation	5.11 (1.21; 6)	5.11 (1.21; 6)	0.818	.001
Registration	2.96 (0.19; 3)	2.88 (0.32; 3)	−0.102	.39
Attention	4.79 (0.92; 5)	4.75 (0.96; 5)	0.689	.001
Calculation	2.20 (0.97; 2)	2.03 (1.00; 2)	0.993	.001
Language	9.58 (1.01; 9)	8.88 (1.28; 9)	0.533	.001
Recall	2.80 (0.40; 3)	2.68 (0.58; 3)	0.691	.001

a Correlation of the total scores between versions: Spearman ρ=0.837 (*P*<.001).

### Phase III: Full Study Implementation

A total of 198 participants were classified into 3 diagnostic groups: dementia (n=44), mild cognitive impairment (n=58), and cognitively normal (n=96). This total was lower than the planned 246 from the prevalence-based calculation. The median age differed significantly across the groups (*P*<.001). Participants with dementia were older (median 73, IQR 69.25‐79 years) and had the highest proportion with only a primary education (30/44, 68.2%). Cognitive and functional measures differed significantly across all assessments (*P*<.001). Median e-TMSE scores were lowest in the dementia group (18.5, IQR 12.0‐22.0) and highest in the cognitively normal group (26.0, IQR 23.0‐28.0). The scores for the Clox test and the category VFT showed a similar pattern. Table 3 presents the baseline participant characteristics for the full implementation sample.

**Table 3.** Baseline demographics and clinical characteristics, full implementation sample (n=198).

Participant characteristics	Dementia (n=44)	MCI^a^ (n=58)	Normal cognition (n=96)	Total (n=198)	*P* value
Age (years), median (IQR)	73 (69.25-79)	69.5 (62.75-75)	65.5 (52-74)	69.5 (59-76)	<.001
Female, n (%)	32 (72.7)	36 (62.1)	69 (71.9)	137 (69.2)	.38
Formal education level, n (%)					
Primary	30 (68.2)	19 (32.8)	36 (37.5)	85 (42.9)	
Secondary	4 (9.1)	8 (13.8)	13 (13.5)	25 (12.6)	
Vocational	3 (6.8)	5 (8.6)	9 (9.4)	17 (8.6)	
Bachelor’s degree	5 (11.4)	22 (37.9)	30 (31.3)	57 (28.8)	
Postgraduate/graduate	2 (4.5)	4 (6.9)	8 (8.3)	14 (7.1)	
e-TMSE^b^, median (IQR)	18.5 (12.0-22.0)	25.0 (23.0-28.0)	26.0 (23.0-28.0)	24 (20-28)	<.001
Clox^c^ test, median (IQR)	3.0 (0.0-8.0)	9.0 (7.0-10.0)	10.0 (8.0-10.0)	9 (6-10)	<.001
Verbal fluency test, median (IQR)	7.0 (5.0-12.8)	14.0 (9.0-19.0)	17.5 (13.0-17.5)	15 (9-19)	<.001
Basic ADLs^d^, median (IQR)	8.0 (3.0-14.8)	0.0 (0.0-2.0)	0.0 (0.0-0.0)	0 (0-3)	<.001
Cognitive IADLs^e^, median (IQR)	8.0 (3.0-12.0)	0.0 (0.0-2.0)	0.0 (0.0-0.0)	0 (0-2)	<.001
IQCODE-16^f^, median (IQR)	4.3 (3.9-4.9)	3.3 (3.1-3.8)	3.3 (3.0-3.3)	3.4 (3.1-3.9)	<.001

a MCI: mild cognitive impairment.

b e-TMSE: electronic Thai Mental State Examination.

c Clox: digitized clock drawing.

d ADLs: activities of daily living.

e IADLs: instrumental activities of daily living.

f IQCODE-16: 16-item Informant Questionnaire on Cognitive Decline in the Elderly.

### Digital Cognitive Test: e-TMSE and Cognitive Assessment

**Figure 6.**
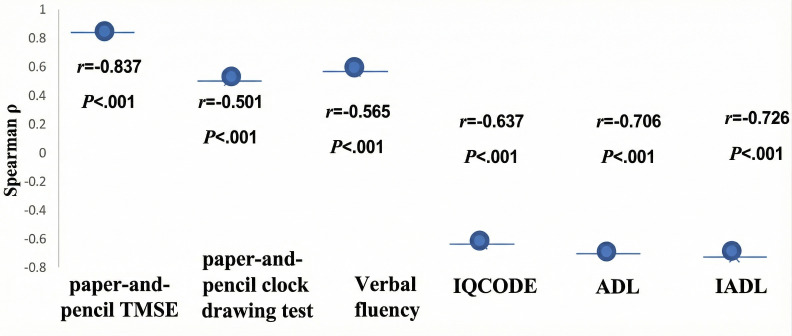
Correlation matrix showing the correlations between the electronic Thai Mental State Examination (e-TMSE) and other cognitive and functional measures, including the Clox test as the paper-and-pencil clock drawing test, the category verbal fluency test, 16-item Informant Questionnaire on Cognitive Decline in the Elderly (IQCODE-16), activities of daily living (ADLs), and cognitive instrumental activities of daily living (IADLs).

Figure 6 illustrates the correlation matrix showing associations between the e-TMSE and related cognitive and functional assessments. Positive correlations were observed between the e-TMSE and the paper-and-pencil CDT (*r*=0.501) and the category VFT (*r*=0.565), indicating convergence with other cognitive measures. In contrast, negative correlations were found between the e-TMSE and informant-reported or functional measures, including the IQCODE-16 (*r*=−0.637) and the ADL score (*r*=−0.706). These patterns reflect inverse relationships between cognitive performance and functional independence.

### Analysis of Cognitive and Functional Assessments for Dementia Screening

**Figure 7.**
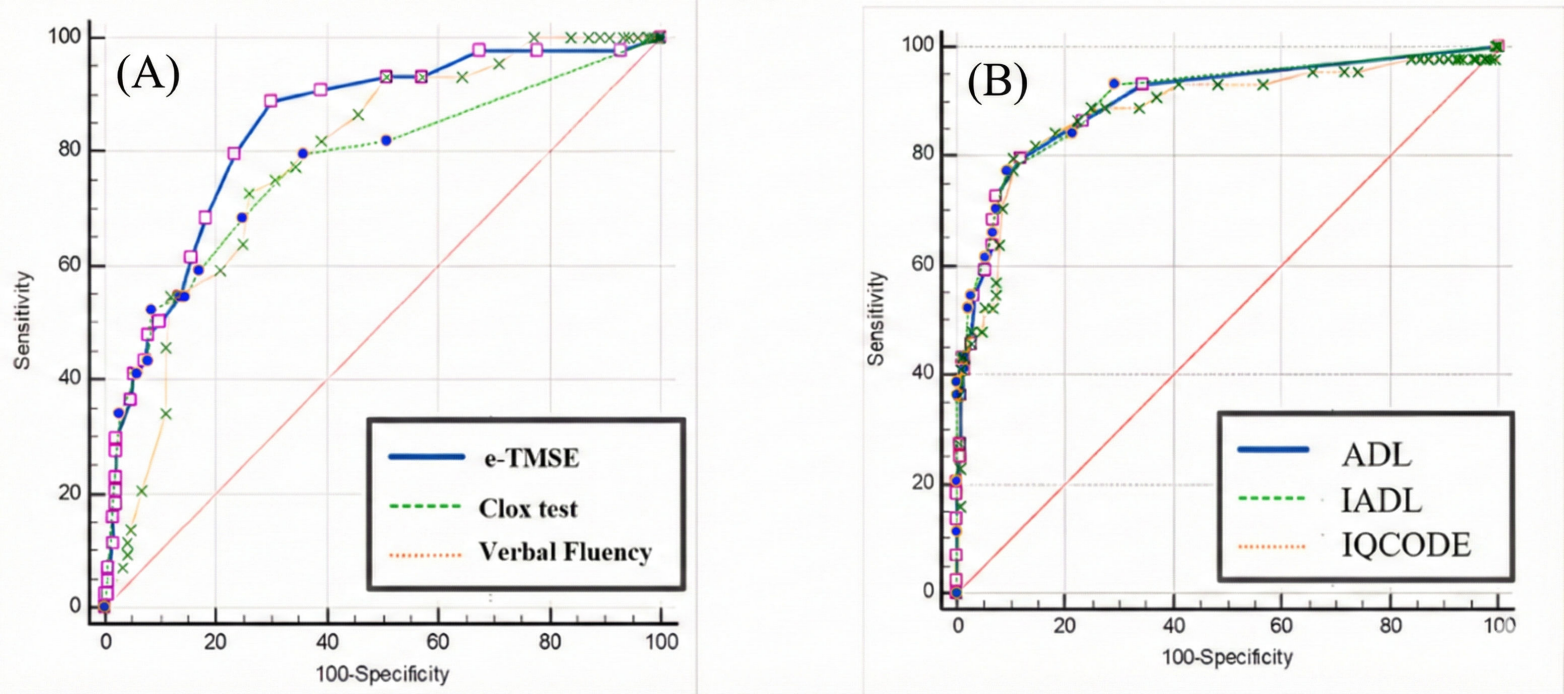
Receiver operating characteristic (ROC) curves for digital measures distinguishing major neurocognitive disorder: (A) electronic Thai Mental State Examination (e-TMSE), Clox test, and category verbal fluency test and (B) activites of daily living (ADLs), cognitive instrumental activities of daily living (IADLs), and 16-item Informant Questionnaire on Cognitive Decline in the Elderly (IQCODE-16). The area under the curve (AUC) values, 95% CIs, and operating thresholds appear in Table 4.

We conducted ROC analyses to evaluate the web-based application’s diagnostic accuracy for distinguishing individuals with normal cognition from those with major neurocognitive disorder. Figure 7 shows the ROC curves, and Table 4 summarizes the performance metrics. The AUC values were as follows: e-TMSE, 0.842; Clox test, 0.774; category VFT, 0.785; IQCODE-16, 0.884; ADL, 0.901; and cognitive IADLs, 0.906. Among these, cognitive IADLs and ADL had the highest discriminatory power. The e-TMSE yielded an optimal cutoff score of ≤23, with a sensitivity of 88.64% and a specificity of 70.13%. The details are provided in Table 4.

Of the 198 participants, 24 (12.1%) were 80 years old or older (9 dementia, 3 mild cognitive impairment, 12 controls); 16 (66.7%) of these 24 individuals had only a primary education. Approximately 5% to 10% of the total cohort (primarily older adults and those with lower education) required assistance but completed the assessment satisfactorily with medical supervision. Errors were most common in drawing tasks for which finger or stylus use was advised. Dropout rates were consistent across sites (5%‐10%). Satisfaction scores (Table S1 in Multimedia Appendix 1) and domain variations that were likely attributable to sociodemographic factors (Tables S2-S3 in Multimedia Appendix 1) are provided in Multimedia Appendix 1.

**Table 4.** Diagnostic performance of *Healthy Brain Test* digital measures for major neurocognitive disorder.

Test	Maximum score	AUROC^a^ (95% CI)	Optimal cutoff	Sensitivity (%)	Specificity (%)	Positive LR^b^	Negative LR
e-TMSE^c^	30	0.842 (0.784–0.890)	≤23	88.64	70.13	2.97	0.16
Clox^d^ test	10	0.774 (0.710-0.830)	≤7	68.17	75.32	2.76	0.42
Verbal fluency test	—^e^	0.785 (0.721-0.840)	≤11	70.73	74.03	2.8	0.37
IQCODE-16^f^	—	0.884 (0.831-0.925)	>3.562	88.64	75.32	3.59	0.15
Basic ADLs^g^	—	0.901 (0.850-0.939)	>3	72.73	92.86	10.18	0.29
Cognitive IADLs^h^	—	0.906 (0.857-0.943)	>1	84.09	75.32	3.59	0.15

a AUROC: area under the receiver operating characteristic curve.

b LR: likelihood ratio.

c e-TMSE: electronic Thai Mental State Examination.

d Clox: digitalized clock drawing.

e Not applicable.

f IQCODE-16: 16-item Informant Questionnaire on Cognitive Decline in the Elderly.

g ADLs: activities of daily living.

h IADLs: instrumental activities of daily living.

## Discussion

### Principal Findings

Web-based cognitive screening applications are a promising approach for early detection of cognitive impairment, particularly in resource-limited settings such as Thailand. We evaluated the feasibility and diagnostic accuracy of digitizing established neuropsychological assessments—the TMSE, clock drawing, verbal fluency, IQCODE-16, and ADL functional evaluations—into a web-based format. Our findings suggest that these web-based tools can reliably identify cognitive impairment and provide screening performance comparable to traditional paper-and-pencil methods.

The TMSE, culturally adapted from the MMSE, is widely used in Thailand as a standard cognitive screening tool for dementia. It evaluates orientation, attention, memory, language, and visuospatial abilities, and prior validation studies reported 85% to 90% sensitivity and 80% to 90% specificity for detecting dementia in Thai populations [15].

This study developed and evaluated a web-based TMSE adaptation (e-TMSE) that preserved the original assessment structure and scoring criteria. Using a diagnostic cutoff of ≤23, the e-TMSE achieved an AUC of 0.84 (95% CI 0.78‐0.89), indicating a high level of discriminative ability for identifying dementia. Sensitivity and specificity were 88.64% and 70.13%, respectively, and were comparable to those of the traditional TMSE. In addition, e-TMSE scores correlated significantly with paper-and-pencil TMSE scores (*r*=0.837, *P*<.001), supporting the concurrent validity of the digital format. Subdomain analyses showed comparable diagnostic performance across most cognitive components, although slight discrepancies were noted in registration and language. These discrepancies may reflect user interaction with the digital interface, particularly among participants with limited touchscreen experience.

The CDT is a brief, effective screening tool for visuoconstructional skills and executive function, which are commonly impaired in dementia. Prior studies reported 76% to 85% sensitivity and 81% to 86% specificity for detecting dementia [22]. In our web-based adaptation, the Clox test achieved an AUC of 0.77 (95% CI 0.71‐0.83), with a cutoff of ≤7; sensitivity was 68.17%, and specificity was 75.32%. Although these values are somewhat lower than some traditional CDT benchmarks, they indicate moderate diagnostic accuracy and support digital screening utility. Performance differences may reflect technological literacy, touchscreen familiarity, and fine motor skills, which can influence task execution and response accuracy in older adults.

The VFT assesses executive function and lexical retrieval. Our implementation used the category (semantic) variant and resulted in an AUC of 0.79 (95% CI 0.72‐0.84) with a cutoff of ≤11. Sensitivity was 70.73%, and specificity was 74.03%, indicating moderate diagnostic accuracy. Semantic fluency, particularly animal naming, is especially useful for early detection of cognitive impairment. Monsch et al [23] reported 100% sensitivity and 92.5% specificity for semantic fluency in distinguishing patients with Alzheimer disease from control participants who had normal cognition. Similarly, a meta-analysis by Henry et al [24] found that semantic fluency declines early in Alzheimer disease and vascular cognitive impairment, reinforcing its role as an early diagnostic marker. Furthermore, studies with individuals with mild cognitive impairment have shown that semantic fluency can be a sensitive and efficient screening tool for early stages of dementia [18,25]. In addition to its diagnostic value, the VFT is simple to administer, cost-effective, and readily adaptable to digital formats. These features make it suitable for large-scale screening and for use in primary care or community-based settings.

The IQCODE provides complementary dementia screening by capturing caregiver-reported longitudinal cognitive change and by circumventing patient insight deficits, which are common in dementia. Previous research reported 80% to 90% sensitivity and specificity for distinguishing dementia from normal aging [26,27]. In our study, the short-form IQCODE (IQCODE-16) showed a significant negative correlation with e-TMSE scores (*r*=−0.637, *P*<.001), consistent with earlier IQCODE and TMSE findings (*r*=−0.679, *P*<.001) [19]. This consistency supports the concurrent validity of the IQCODE-16 as a reliable screening instrument. IQCODE-16 achieved an AUC of 0.88 (95% CI 0.83‐0.93), with a cutoff of >3.562. Sensitivity was 88.64%, and specificity was 75.32%, with a marginally higher AUC than e-TMSE (AUC=0.84). These findings highlight clinical relevance, particularly in community-based or resource-limited settings where cultural, educational, or language barriers may affect performance-based testing.

Functional assessments, especially ADL, provide essential context regarding the real-world impact of cognitive decline on daily functioning. Although not diagnostic in isolation, declines in ADL—particularly cognitive IADLs—are predictive of dementia progression. Our cognitive IADL component achieved an AUC of 0.91 (95% CI 0.86‐0.94), with a cutoff of >1; sensitivity was 84.09%, and specificity was 75.32%. The ADL measure achieved an AUC of 0.90 (95% CI 0.85‐0.94), with a cutoff of >3; sensitivity was 72.73%, and specificity was 92.86%.

Adapting neuropsychological and functional assessments to web-based platforms, such as *Healthy Brain Test*, represents meaningful progress in dementia screening. These platforms can expand access in resource-limited regions and facilitate earlier detection of cognitive decline. In Thailand, geographic and socioeconomic disparities limit access to specialized care, and a culturally adapted, validated digital tool may help bridge screening gaps in community-based settings. In our findings, dropouts were most common among the oldest-old and those with lower education levels, primarily due to unfamiliarity with digital technology. Issues also arose from misaligned user expectations; for instance, participants often wished to erase specific lines during drawing tasks, whereas the application only permitted clearing the entire page to redraw. Additional factors contributing to dropout included poor user interface design (eg, small font sizes, login difficulties) and physical health limitations (eg, visual impairment, hand tremors). Our study recruited individuals with diverse baseline characteristics regarding age, education, and diagnostic status (dementia, mild cognitive impairment, and normal controls). It is well-established that economic conditions differ regionally in Thailand; generally, the central, southern, and northern regions are wealthier and report better health outcomes than the northeast. These socioeconomic and cultural variations may explain some of the observed differences in cognitive performance, both within the normal group and across the entire cohort at each study site.

The global adoption of mobile and web-based cognitive screening tools has expanded, offering promising alternatives to traditional paper-and-pencil assessments across clinical and community settings. The commercially available *BrainTest* reported 71% sensitivity and 90% specificity compared with comprehensive neuropsychological assessments [28]. Similarly, *M-CogScore*, which includes memory, attention, and executive tasks, achieved an AUC of 0.85 (95% CI 0.80‐0.91) and outperformed the MMSE-2 (AUC=0.78, 95% CI 0.72‐0.84) for distinguishing individuals with normal cognition from those with impairment [29]. In parallel, mobile cognitive screening tools designed for smartphones and tablets provide additional advantages over traditional modalities [30].

Nevertheless, important limitations persist. Previous studies [30,31] noted that most self-administered electronic tools lack rigorous psychometric validation. Practical barriers include touchscreen usability challenges among older adults with limited digital experience, as well as broader issues of digital literacy. In addition, digital tools may be less able to detect subtle behavioral, emotional, or motivational cues that inform clinical assessment. These limitations are especially pronounced in resource-limited settings, where disparities in technology access and health services can further hinder effectiveness.

In Thailand, electronic cognitive assessments have been used for over a decade. A 2018 validation study of the computerized TMSE [32] demonstrated a strong correlation with the standard pen-and-paper TMSE. The percentage of agreement between the two methods was 92.5%, with a Kappa coefficient of 0.85 (*P*<.001). The duration of the standard pen-and-paper TMSE was slightly shorter than that of the computerized TMSE (7.31 min vs 7.97 min; 95% CI −1.159 to −0.175; *P*=.09). More recently, a study on the Thai electronic MoCA [33] reported a high correlation for total scores, with a concordance correlation coefficient of 0.919 and a mean difference of −0.204 (95% CI −6.311 to 5.904). All cognitive subdomain scores showed moderate to high concordance correlation coefficient values exceeding 0.4. Furthermore, functional assessment is crucial for determining the stage of cognitive performance. Therefore, it is essential to evaluate both cognitive function and ADLs concurrently. ADLs can be self-reported or observed by family members who can provide feedback to clinicians. In the context of Thai culture, where extended families are common, family members play a vital role in observing daily functioning and providing this necessary feedback.

Thailand’s cultural, linguistic, and socioeconomic factors require careful consideration when implementing web-based cognitive screening tools. Our study showed that *Healthy Brain Test*, a web-based cognitive assessment application, is clinically useful for the early detection of dementia. The tool demonstrated good psychometric performance and was validated across diverse Thai populations in multiple regions, strengthening the applicability of the findings.

Several limitations warrant consideration. The sample comprised mostly individuals with normal cognition, mild cognitive impairment, and mild dementia, with limited evaluation of performance in moderate to severe dementia. Most notably, the final sample size was lower than the planned target of 246, which may have reduced the precision of estimates. However, a 20% prevalence of dementia with a 95% CI and with a margin of error of 5.6% led to the sample size of 198 participants in our study. Assessments were conducted under professional supervision to ensure understanding in the local Thai pronunciation, which may not reflect real-world, unsupervised use. However, caregivers can possibly play this role by helping individual older adults at home. Further limitations of our study primarily concern digital literacy and accessibility. First, a lack of digital experience was evident; the majority of older Thai participants were unfamiliar with drawing on touchscreens (eg, tablets or smartphones). However, they were able to perform tasks correctly after receiving instruction. In the context of unsupervised community testing, co-resident children or visiting family members can play a crucial role in encouraging older adults to adopt new technologies. For example, the Thai medical system successfully implemented telemedicine during the COVID-19 era, and hospitals continue to use it for patients in remote areas. A key factor in this success is the assistance provided by younger family members during assessments. Second, regarding physical and sensory impairments, Thailand’s universal health coverage scheme allows individuals with vision or hearing problems to receive fully reimbursed treatments, such as cataract extraction with lens implantation and hearing aids. Third, regarding the need for technological support, older adults with cognitive impairment may require caregivers to set up or assist with web-based assessments. Despite this support, 5% to 10% of our cohort were unable to complete the assessment. Fourth, socioeconomic factors such as cost, income level, and access to reliable internet and devices may pose barriers. To address this, the Thai government has set a goal to establish free internet access zones in every village, and digital devices imported from China are becoming increasingly affordable. Last, technological anxiety remains a common issue among older adults.

Although our study included a relatively small sample size, it was conducted across 4 distinct provinces: Bangkok (Siriraj and Vajira Hospitals), Phitsanulok (Northern Thailand), Nakhon Phanom (Northeastern Thailand, bordering the Mekong River and Laos), and Phatthalung (Southern Thailand). We recently signed a Memorandum of Understanding with the Department of Medical Services, Ministry of Public Health, to deploy this web-based cognitive screening test nationwide over the next 3 years. Based on the insights gained from this widespread implementation, we anticipate developing a second iteration or an abbreviated version of the assessment.

Future research should evaluate *Healthy Brain Test* in unsupervised community-based settings, explore integration with digital health infrastructure, and enable longitudinal tracking of cognitive change. Implementation should be supported by health care professionals to ensure appropriate interpretation of results and timely referral for further evaluation or intervention.

### Conclusion

This study demonstrates that the *Healthy Brain Test* web-based application is valid, efficient, and accessible for early detection of cognitive impairment. The battery comprises the following assessments: e-TMSE, CDT, category VFT, IQCODE-16, and ADL. The digital test scores correlated strongly with traditional paper-and-pencil assessments, supporting the reliability and clinical applicability of our web-based cognitive screening application. This simple, rapid tool is well-suited for identifying cognitive impairment in primary care and community-based settings. Further studies should validate performance in unsupervised and diverse populations and support integration into routine cognitive health screening protocols.

## Supplementary material

10.2196/85454 Multimedia Appendix 1 Satisfaction scores and site-specific findings.

## Abbreviations

ADL: activities of daily living
AUC: area under the curve
CDT: clock drawing test
DSM-V: Diagnostic and Statistical Manual of Mental Disorders, Fifth Edition
e-TMSE: electronic Thai Mental State Examination
IADL: instrumental activities of daily living
IQCODE: Informant Questionnaire on Cognitive Decline in the Elderly
MMSE: Mini-Mental State Examination
MoCA: Montreal Cognitive Assessment
ROC: receiver operating characteristic
TMSE: Thai Mental State Examination
VFT: verbal fluency test
