# Supplementary material for: Web-Based Application for Cognitive and Functional Assessments in Dementia Screening: Mixed Methods, User-Centered Development Approach
Source: JMIR Hum Factors. 2026 Feb 19;13:e85454. doi: 10.2196/85454 (PMC12919909; doi:10.2196/85454)
Supplement: Multimedia Appendix 1 [file humanfactors-v13-e85454-s001.docx]

**Supplementary**

| **Table S1.** Evaluation results of user satisfaction toward the web application. | | | |
| --- | --- | --- | --- |
| **Domains and Items** | **Mean** | **S.D.** | **Interpretation** |
| ***1. Web Application Design*** |  |  |  |
| 1.1 Smoothness of operation | 3.81 | 0.84 | Good |
| 1.2 Speed of text and image loading | 3.90 | 0.82 | Good |
| 1.3 Appropriateness of screen layout | 3.92 | 0.97 | Good |
| 1.4 Legibility of images and text | 4.17 | 0.88 | Good |
| 1.5 Appropriateness of language and audio | 4.06 | 0.89 | Good |
| **2. Web Application Usage** |  |  |  |
| *2.1 Ease of use* | 3.77 | 0.90 | Good |
| 2.2 User data privacy protection | 3.87 | 0.91 | Good |
| 2.3 Appropriateness of user interaction | 3.96 | 0.84 | Good |
| 2.4 Presentation of final test results | 4.12 | 0.76 | Good |
| 2.5 Speed of answer verification | 4.10 | 0.77 | Good |
| 2.6 Utility for dementia screening | 4.19 | 0.72 | Good |
| 2.7 Modernity of the web application | 4.25 | 0.71 | Good |
| ***3. Overall Satisfaction*** | 4.04 | 0.66 | Good |
| The mean scores were interpreted to determine the level of user satisfaction using the following criteria: 4.50 – 5.00: Excellent, 3.50 – 4.49: Good, 2.50 – 3.49: Moderate, 1.50 – 2.49: Poor and 1.00 – 1.49: Very Poor | | | |
|  |  |  |  |
|  |  |  |  |

| **Table S2.** Comparison of cognitive and functional assessment scores across five study sites. | | | | | | |
| --- | --- | --- | --- | --- | --- | --- |
| **Study sites** | **BKK Siriraj** | **BKK Vajira** | **Phatthalung** | **Nakhon Phanom** | **Phitsanulok** | **p-value** |
|  | **n=77** | **n=8** | **n=57** | **n=42** | **n=14** |  |
| **Age (years)** | 69.0 (63.0-75.0) | 76.0 (66.8-82.5) | 71.0 (56.0-77.0) | 67.0 (56.5-76.3) | 59.0 (46.5-66.3) | 0.012 |
| **Female (%)** | 51 (66.2%) | 5 (62.5%) | 39 (68.4%) | 30 (71.4%) | 12 (85.7%) | 0.664 |
| **Education** |  |  |  |  |  | 0.009 |
| **Primary** | 24 (31.2%) | 1 (12.5%) | 33 (57.9%) | 23 (54.8%) | 4 (28.6%) |  |
| **Secondary** | 9 (11.7%) | 1 (12.5%) | 5 (8.8%) | 7 (16.7%) | 3 (21.4%) |  |
| **Vocational** | 11 (14.3% | 0 (0.0%) | 1 (1.8%) | 3 (7.1%) | 2 (14.3%) |  |
| **Bachelor's degree** | 26 (33.8%) | 6 (75.0%) | 13 (22.8%) | 9 (21.4%) | 3 (21.4%) |  |
| **Postgraduate/Graduate** | 7 (9.1%) | 0 (0.0%) | 5 (8.8%) | 0 (0.0%) | 2 (14.3%) |  |
| **e-TMSE** | 25.0 (23.0-28.0) | 23.0 (21.3-25.8) | 24.0 (20.0-28.0) | 18.5 (12.8-24.3) | 26.5 (25.0-29.0) | <0.001 |
| **e-clox** | 10.0 (7.0-10.0) | 8.0 (2.5-10.0) | 9.0 (8.0-10.0) | 4.0 (0.0-9.3) | 10.0 (8.8-10.0) | <0.001 |
| **e-Verbal Animal** | 16.48 (13.0-20.0) | 5.0 (2.0-5.0) | 13.0 (8.5-20.0) | 12.0 (5.8-18.0) | 16.5 (11.5-21.0) | <0.001 |
| **e-ADL** | 0.0 (0.0-2.5) | 1.5 (0.0-5.3) | 1.0 (0.0-2.0) | 1.5 (0.0-12.3) | 0.0 (0.0-0.0) | 0.003 |
| **e-iADL** | 0.0 (0.0-2.0) | 1.0 (0.0-4.5) | 0.0 (0.0-2.0) | 1.0 (0.0-12.0) | 0.0 (0.0-0.0) | 0.003 |
| **e-IQCODE** | 3.3 (3.1-3.8) | 3.2 (2.5-3.9) | 3.4 (3.2-3.9) | 3.7 (3.2-4.8) | 3.2 (3.0-3.3) | 0.004 |
| Data are presented as Median (Interquartile Range). P-values indicate significant differences between groups (Kruskal-Wallis test). Abbreviations: e-TMSE = electronic Thai Mental State Examination; e-clox = electronic Clock Drawing Test; e-ADL = electronic Activities of Daily Living; e-IQCODE = electronic Informant Questionnaire on Cognitive Decline in the Elderly. | | | | | | |
|  |  |  |  |  |  |  |

| **Table S3.** Cognitive and functional performance of the healthy control group stratified by study site. | | | | | |
| --- | --- | --- | --- | --- | --- |
| **Study sites** | **BKK Siriraj** | **Phatthalung** | **Nakhon Phanom** | **Phitsanulok** | **p-value** |
|  | **n=33** | **n=39** | **n=22** | **n=2** |  |
| **Age (years)** | 66.0 (56.5-72.0) | 70.0 (51.0-78.0) | 57.0 (48.0-73.0) | 43.5 (32.0-) | 0.132 |
| **Female (%)** | 22 (66.7%) | 28 (71.8%) | 17 (77.3%) | 2 (100.0%) | 0.672 |
| **Education** |  |  |  |  | 0.049 |
| **Primary** | 5 (15.2%) | 21 (53.8%) | 10 (45.5%) | 0 (0.0%) |  |
| **Secondary** | 4 (12.1%) | 4 (10.3%) | 4 (18.2%) | 1 (50.0%) |  |
| **Vocational** | 5 (15.2%) | 1 (2.6%) | 3 (13.6%) | 0 (0.0%) |  |
| **Bachelor's degree** | 15 (45.5%) | 9 (23.1%) | 5 (22.7%) | 1 (50.0%) |  |
| **Postgraduate/Graduate** | 4 (12.1%) | 4 (10.3%) | 0 (0.0%) | 0 (0.0%) |  |
| **e-TMSE** | 28.0 (25.5-29.0) | 26.0 (20.0-28.0) | 24.0 (17.3-26.3) | 27.5 (27.0-) | 0.003 |
| **e-clox** | 10.0 (9.5-10.0) | 9.0 (8.0-10.0) | 7.5 (4.0-10.0) | 10.0 (10.0-10.0) | 0.005 |
| **e-Verbal Animal** | 19.0 (15.5-22) | 17.0 (9.0-21.0) | 16.0 (12.0-19.0) | 21.5 (18.0-) | 0.047 |
| **e-ADL** | 0.0 (0.0-0.0) | 0.0 (0.0-2.0) | 0.0 (0.0-1.3) | 0.0 (0.0-0.0) | 0.289 |
| **e-iADL** | 0.0 (0.0-0.0) | 0.0 (0.0-2.0) | 0.0 (0.0-1.0) | 0.0 (0.0-0.0) | 0.121 |
| **e-IQCODE** | 3.1 (3.0-3.3) | 3.3 (3.1-3.7) | 3.3 (3.1-3.6) | 1.8 (1.8-) | 0.006 |
| Data are presented as Median (Interquartile Range). P-values indicate significant differences between groups (Kruskal-Wallis test). Abbreviations: e-TMSE = electronic Thai Mental State Examination; e-clox = electronic Clock Drawing Test; e-ADL = electronic Activities of Daily Living; e-IQCODE = electronic Informant Questionnaire on Cognitive Decline in the Elderly. | | | | | |
|  |  |  |  |  |  |
